# Autism symptoms in anorexia nervosa: a comparative study with females with autism spectrum disorder

**DOI:** 10.1186/s13229-021-00455-5

**Published:** 2021-06-30

**Authors:** Jess Kerr-Gaffney, Hannah Hayward, Emily J. H. Jones, Daniel Halls, Declan Murphy, Kate Tchanturia

**Affiliations:** 1 Department of Psychological Medicine, Institute of Psychiatry, Psychology, and Neuroscience, King’s College London, 103 Denmark Hill, London, SE5 8AZ UK; 2 Department of Forensic and Neurodevelopmental Sciences, Institute of Psychiatry, Psychology, and Neuroscience, King’s College London, London, UK; 3 Centre for Brain and Cognitive Development, Birkbeck, University of London, London, UK; 4 Psychological Medicine Clinical Academic Group, National Eating Disorders Service, South London and Maudsley NHS Trust, London, UK; 5 Department of Psychology, Ilia State University, Tbilisi, Georgia

**Keywords:** Anorexia nervosa, Autism spectrum disorder, Comorbidity, Diagnosis, Autism diagnostic observation schedule, Screening

## Abstract

**Background:**

Recent research suggests a link between autism spectrum disorder (ASD) and anorexia nervosa (AN). Individuals with AN show high scores on measures of ASD symptoms, relative to individuals without AN, however, there are currently no studies directly comparing women with AN to women with ASD. The aim of the current study was to examine profiles of ASD symptoms in young women in the acute and recovered stages of AN, women with ASD, and typically developing controls (TD), on both self-report and clinical interview measures.

**Methods:**

Four groups of participants aged 12–30 years were included (*n* = 218): AN, recovered AN (REC), ASD, and TD. Group differences on the Social Responsiveness Scale, 2nd edition (SRS-2), 10-item Autism Quotient (AQ-10), and the Autism Diagnostic Observation Schedule, 2nd edition (ADOS-2) were examined. To explore similarities and differences in specific symptom profiles associated with AN and ASD, individual item endorsement on the ADOS-2 was also examined in AN, REC, and ASD.

**Results:**

Across measures, women with ASD showed the highest scores, and TDs the lowest. Generally, individuals with AN and REC showed intermediate levels of ASD symptoms, scoring between the other two groups. However, AN and ASD did not differ on restricted interests and repetitive behaviour subscales. The ADOS-2 item ‘quality of social response’ adequately discriminated between ASD and non-ASD participants.

**Limitations:**

A full diagnostic assessment for ASD was not provided for participants with AN/REC, nor were eating disorders assessed in the ASD group. Therefore, some diagnostic overlap between groups is possible. The cross-sectional design is another limitation.

**Conclusions:**

The results suggest similarities in scores on both self-report and clinical interview measures in AN and ASD. However, individual ADOS-2 item analyses also revealed subtle differences, particularly in reciprocal social interaction. ASD symptoms may be a combination of both state and trait features in AN.

**Supplementary Information:**

The online version contains supplementary material available at 10.1186/s13229-021-00455-5.

## Introduction

Anorexia nervosa (AN) is a severe eating disorder characterised by an intense fear of weight gain, persistent behaviour to restrict energy intake, and a disturbance in the way one’s body weight or shape is experienced [1]. AN is more common in females, with a male to female sex ratio of 1: 10, although epidemiological studies have reported ratios as low as 1: 3, likely reflecting clinical under detection in males [2]. AN usually emerges in adolescence, with a peak age of onset between 15 and 19 years [3]. While several biological, psychological, and temperamental factors are thought to be implicated in the development and maintenance of the disorder, no single psychological or pharmacological intervention has proven to be particularly effective in treating AN [4, 5]. On the other hand, autism spectrum disorder (ASD) is a life-long neurodevelopmental disorder associated with persistent difficulties in social communication and interaction, as well as restricted and repetitive behaviours and interests [1]. ASD affects around 1% of the population and symptoms are typically recognised during early childhood [6]. While ASD has historically been viewed as a predominantly male disorder, there has been growing recognition of the female presentation of ASD, with population-based studies estimating a more modest male to female sex ratio of 3: 1 [7]. Gender differences in symptom presentation, camouflaging, and stereotyped understandings of ASD in professionals, parents, and teachers are all thought to contribute to underdiagnosis of females with ASD [8–11].

Research has accumulated to suggest a relationship between AN and ASD. For example, similarities in neuropsychological and social-cognitive functioning have been reported, including poor set shifting performance [12], weak central coherence [13, 14], superior attention to detail [15, 16] and difficulties in theory of mind [17], empathy [18, 19], and emotion recognition [20, 21]. Individuals with AN also report high levels of social anxiety, isolation, and difficulties in peer relationships and bullying, experiences which mirror those of women with ASD [22–24]. These similarities suggest possible overlap in the mechanisms that contribute to the development of ASD and AN, with some even suggesting that AN is a female manifestation of ASD [25]. A key question is whether AN and ASD are indeed linked aetiologically, or whether starvation or other factors associated with the acute state of AN are producing autistic-like symptoms, such as behavioural rigidity and social withdrawal.

At the behavioural level, individuals with AN show high levels of ASD symptoms on both self-report and clinical interview measures of ASD. For example, around one third of those with AN score above the clinical cut-off on the Autism Diagnostic Observation Schedule, 2nd edition (ADOS-2) [26]. The ADOS-2 is considered a “gold-standard” assessment of current ASD symptoms recommended as part of diagnostic assessments, along with the Autism Diagnostic Interview-Revised (ADI-R) to assess developmental history [27]. Similar rates are reported in individuals recovered from AN [28, 29], suggesting that ASD symptoms are not merely side-effects of starvation. Similarly, those with ASD show significantly more eating disorder symptoms than non-autistic people, with around 27% of women with ASD reporting clinically significant levels of eating disorder symptoms [30–32]. Eating difficulties are also very common in children with ASD, particularly food selectivity and avoidant/restrictive food intake disorder [33]. Consistent with the hypothesis that ASD may be a risk factor for AN, evidence suggests that higher autistic symptoms in childhood predict increased disordered eating in adolescence [34]. However, other longitudinal studies have failed to find a relationship between early ASD symptoms and AN in adolescence [35, 36].

Although the aforementioned studies provide evidence of shared symptomatology and comorbidity between the two conditions, few studies have directly compared ASD symptoms in AN and ASD samples. One study found that individuals with ASD and those with AN showed similarly high scores on the Autism Quotient (AQ) [37], and others report significantly higher scores in ASD compared to AN [38, 39]. Importantly, these studies compared a female AN group to a male ASD group, introducing a substantial confound. Recent work has demonstrated differences in symptom presentation in males and females with ASD. For example, women with ASD are less likely to show restricted interests and repetitive behaviours, but show more sensory processing abnormalities than men with ASD [8, 40]. Women are also more likely to camouflage or hide their autistic traits than males, and as a result, show fewer surface-level social difficulties despite similar performance on tasks of social understanding as males with ASD [23, 41]. Given these differences in male and female presentations of ASD, it is essential that studies compare AN and ASD samples matched for sex. This will enable us to understand whether women with AN with high scores on ASD measures show similar patterns of symptoms across domains to women with ASD. On the other hand, it may be that factors such as low body mass index (BMI) or starvation are producing autistic-like symptoms which are inflating scores on ASD measures. Including individuals who are recovered from AN will allow us to establish whether these symptoms represent true ASD comorbidity in AN.

Thus, the aim of the current investigation was to compare ASD symptoms in females in the acute and recovered stages of AN, females with ASD, and a typically developing comparison group (TD), of similar age, sex, and IQ. As women with ASD sometimes camouflage their symptoms, we chose to include measures that capture different aspects of ASD symptoms; both outward behaviour as well as an individual’s self-reported feelings and perceptions of their own behaviour. Specifically, between-group differences on both self-report (10-item Autism Quotient [AQ-10], Social Responsiveness Scale, 2nd edition [SRS-2]) and observational interview measures (ADOS-2) will be examined. Further, individual item endorsement on the ADOS-2 will be examined in order to elucidate similarities and differences in specific symptom profiles in those with AN and those with ASD.

## Methods

### Participants and design

Four groups were included in the study: acute AN, recovered AN (REC), ASD, and TD. Data were extracted from three existing data sets. AN, REC, and TD participants were from two studies investigating social and emotional functioning in AN [28, 42], while participants with ASD were from the European Autism Interventions Longitudinal European Autism Project (EU-AIMS LEAP) [43]. Details regarding participant recruitment for the original studies can be found in the Additional file 1.

Individuals with AN typically show average or above average intelligence [44], and the majority of cases occur within adolescence and young adulthood [3, 45]. In order to ensure similar age and IQ ranges across groups, the following inclusion criteria were therefore applied to all participants: female, aged 12–30 years, with average or above average IQ (≥ 85), and full item-level data available for the main outcome measure (ADOS-2 module 4). Additionally, AN and REC met criteria for current or past AN according to the Structured Clinical Interview for DSM-5 Disorders, research version (SCID-5-RV) [46]. Participants with AN were required to have a BMI ≤ 18.5, while REC a BMI between 18.5 and 27, maintained within this range for at least one year prior to testing. For participants under 18 years, percentage of ideal body weight (%IBW) was used instead of BMI. Participants with AN were required to have a %IBW < 85, and REC participants a %IBW > 85 [47]. TD were screened using the SCID-5-RV to ensure they did not show symptoms consistent with any psychiatric disorders and were not using psychiatric medication. To ensure absence of clinical or sub-clinical eating disorder symptoms, TD with eating disorder examination questionnaire (EDE-Q) scores > 2.7 were not included. TD were also required to have a BMI between 18.5 and 27. Although a BMI of 25 is considered the upper limit of the healthy range, a slightly higher upper limit was chosen for this study to better represent the general female population in England [48]. Participants with ASD met DSM-IV [49], DSM-5 [1], or ICD-10 [50] criteria. ASD diagnoses were based on a comprehensive assessment of the participant’s clinical history and current symptoms.

To reduce diagnostic crossover between groups, any AN and REC participants with an existing diagnosis of ASD were not included (*n* = 6). Information regarding comorbid eating disorder diagnoses was not available for participants with ASD.

### Measures

#### ASD symptoms

The ADOS-2 [51] is a semi-structured interview which includes a range of questions and activities designed to evoke behaviours and cognitions associated with ASD. The revised algorithm, which was designed to more closely reflect the DSM-5 criteria for ASD, was used for scoring [52]. Compared to the original algorithm, the revised algorithm demonstrates greater sensitivity in females, as well as those with average or above average IQ, making it particularly well suited to the current study [53]. It has two subscales: social affect and restricted and repetitive behaviour. The sum of the subscale scores is the total score; with scores of 8 or more indicating possible ASD. Interviews were administered by researchers who had received ADOS-2 training and met requirements for research reliability.

The AQ-10 [54] is a brief self- or parent-report questionnaire for ASD symptoms. The majority of participants in this study took the adult self-report version, however for a small proportion of adolescents with ASD (6% of the total sample), a parent completed the adolescent parent-report version. As both versions contain the same items and use the same clinical cut-off of 6, data from both versions can be analysed together [54].

The SRS-2 [55] is a self-report questionnaire measuring symptoms associated with ASD. Higher scores indicate more severe symptoms. As well as a total score (max 195), there are five subscales: social awareness, social cognition, social communication, social motivation, and restricted interests and repetitive behaviours. Total scores can also be converted to t-scores: ≤ 59 within normal limits; 60–65 mild symptoms; 66–75 moderate symptoms; and ≥ 76 severe symptoms.

#### Comorbid psychopathology and clinical information

The EDE-Q [56] is a self-report questionnaire assessing severity of eating disorder psychopathology in AN, REC, and TD. Total scores are calculated by averaging responses across all items, with higher scores indicating more severe symptoms (max 6). The EDE-Q was not collected in participants with ASD.

The Hospital Anxiety and Depression Scale (HADS) [57] measured anxiety and depression in AN, REC, and TD, while the Beck Anxiety and Depression Inventories (BAI/BDI) [58, 59] or the Beck Youth Inventory, 2nd edition (BYI-II) [60] measured anxiety and depression in participants with ASD. All are self-report questionnaires, where higher scores indicate more severe psychopathology. As different measures were used across studies, Z scores were calculated. For AN and REC, *Z* scores were based on the mean and standard deviation (SD) of the TD group. For participants with ASD, Z scores were based on the mean and SD of the age- and sex-matched TDs included in the EU-AIMS LEAP study.

IQ was measured using the Wechsler Abbreviated Scales of Intelligence-Second Edition (WASI-II) [61] or the National Adult Reading Test (NART) [62] in participants with AN, REC, and TD. In ASD, IQ was assessed using a range of different scales depending on the participants’ age and verbal ability. The majority (68.3%) took the WASI-II, however the Wechsler Adult Intelligence Scale, Revised (WAIS-R), WAIS-III, WAIS-IV, and the Wechsler Intelligence Scale for Children, 3rd edition (WISC-III) were also used in a minority of participants.

Height and weight were measured at the study session to calculate BMI (weight/height^2^).

### Analysis

Histograms and Q–Q plots were inspected to check for normal distributions. Homogeneity was assessed using Levene’s test. Group differences in demographic characteristics, psychopathology, and ASD symptoms (ADOS-2, AQ-10, and SRS-2 total and subscale scores) were assessed with one-way analysis of variance (ANOVA) and Tukey’s post-hoc tests, or Welch’s ANOVA with Games-Howell post-hoc tests where the assumption of homogeneity was violated. The Kruskal–Wallis test was used for non-normally distributed data, with Bonferroni corrected post-hoc tests. Medians and interquartile range (IQR) are reported for such data. Independent samples t-tests were used for demographic variables where only two groups were compared (e.g., age at AN diagnosis). Chi-square tests were used to assess group differences in the proportion of participants scoring above clinical cut-offs on ASD measures, as well as for demographic or clinical measures with a binary outcome (e.g., medication use). Effect sizes are reported for ANOVAs (*ηp*^2^), Kruskal–Wallis tests (*η*^2^), independent samples t tests (Cohen’s d), and Chi-square tests (Cramer’s V).

To examine group differences in individual item endorsement on the ADOS-2 in AN, REC, and ASD, scores on each item were converted to a binary variable. Scores of 1, 2, or 3 (indicating some level of ASD symptomatology) were “endorsed”, while scores of 0 were “not endorsed” (indicating no observable ASD symptoms). Scores of 7 or 8 (e.g., where an item is not codable due to physical disability) were treated as missing data. This method allowed us to separate the severity of symptoms from the occurrence of symptoms in each group. Group differences in the proportion of participants endorsing each item were assessed using Chi-square tests (or Fisher’s exact test where the minimum expected cell count assumption was violated). ADOS-2 items that best discriminated between individuals with AN and ASD were also identified. These were items that were endorsed by at least 66% of participants with ASD, and fewer than 33% of participants with AN. This approach was used in a previous study addressing a similar research question [63]. TD were not included in the item endorsement analyses, as discriminating between ASD and TD, as well as AN and TD using the ADOS-2 was not the purpose of this study and has been covered elsewhere [52, 53, 64]. A significance level of *α* = 0.01 was used across analyses, however no correction was applied to account for multiple comparisons [65]. SPSS statistics v26 [66] was used for analyses.

## Results

In total, 218 participants were included in the study (64 AN, 46 REC, 41 ASD, 67 TD). Demographic and clinical information are provided in Table 1. Groups did not significantly differ in age or IQ. REC and ASD showed similar levels of anxiety and depression, significantly higher than HC, but lower than in AN. The proportion of participants currently taking psychiatric medication was similar across the three clinical groups.

**Table 1:** Mean (SD) demographic and clinical characteristics

	AN (*n* = 64)	REC (*n* = 46)	ASD (*n* = 41)	TD (*n* = 67)	Test statistics	*p*-value	Effect size
Age (years)	21.53 (4.15)	22.21 (3.47)	20.56 (8.42)	22.16 (3.60)	*F*(3,106.95) = 1.21	0.31	0.02
IQ	109.97 (9.97)	110.91 (10.39)	107.31 (10.89)	111.46 (8.42)	*F*(3,208) = 1.60	0.19	0.02
BMI^†^	16.63 (2.60)^a^	21.05 (3.11)^b^	21.60 (5.27)^b^	21.57 (1.70)^b^	*X*^2^(3) = 127.12	** < 0.001**	0.58
%on psychiatric medication	50%^a^	39%^a^	30%^a^	0%^b^	*X*^2^(3) = 43.80	** < 0.001**	0.45
Age at AN diagnosis	16.80 (3.62)	15.56 (2.52)	–	–	*t*(102.89) = 2.08	0.04	0.40
EDE-Q^†^	3.62 (2.60)^a^	1.15 (1.71)^b^	–	0.34 (0.83)^c^	*X*^2^(2) = 82.71	** < 0.001**	0.47
Anxiety (*Z* score)	2.43 (1.42)^a^	1.71 (1.71)^ab^	1.40 (1.93)^b^	0.00 (1.00)^c^	*F*(3,91.87) = 44.50	** < 0.001**	0.31
Depression (*Z* score)	3.79 (2.74)^a^	1.72 (2.01)^b^	2.04 (2.15)^b^	0.00 (1.00)^c^	*F*(3,88.90) = 44.36	** < 0.001**	0.35

AN, anorexia nervosa; ASD, autism spectrum disorder; BMI, body mass index; EDE-Q, eating disorder examination questionnaire; IQ, intelligence quotient; REC, recovered anorexia nervosa; SD, standard deviation; TD, typically developing controls

Different superscripts indicate significant differences between groups, significant *p*-values (< .01) are highlighted in bold

^†^Median and interquartile range (IQR)

### ASD symptoms-scale level

**Fig. 1:**
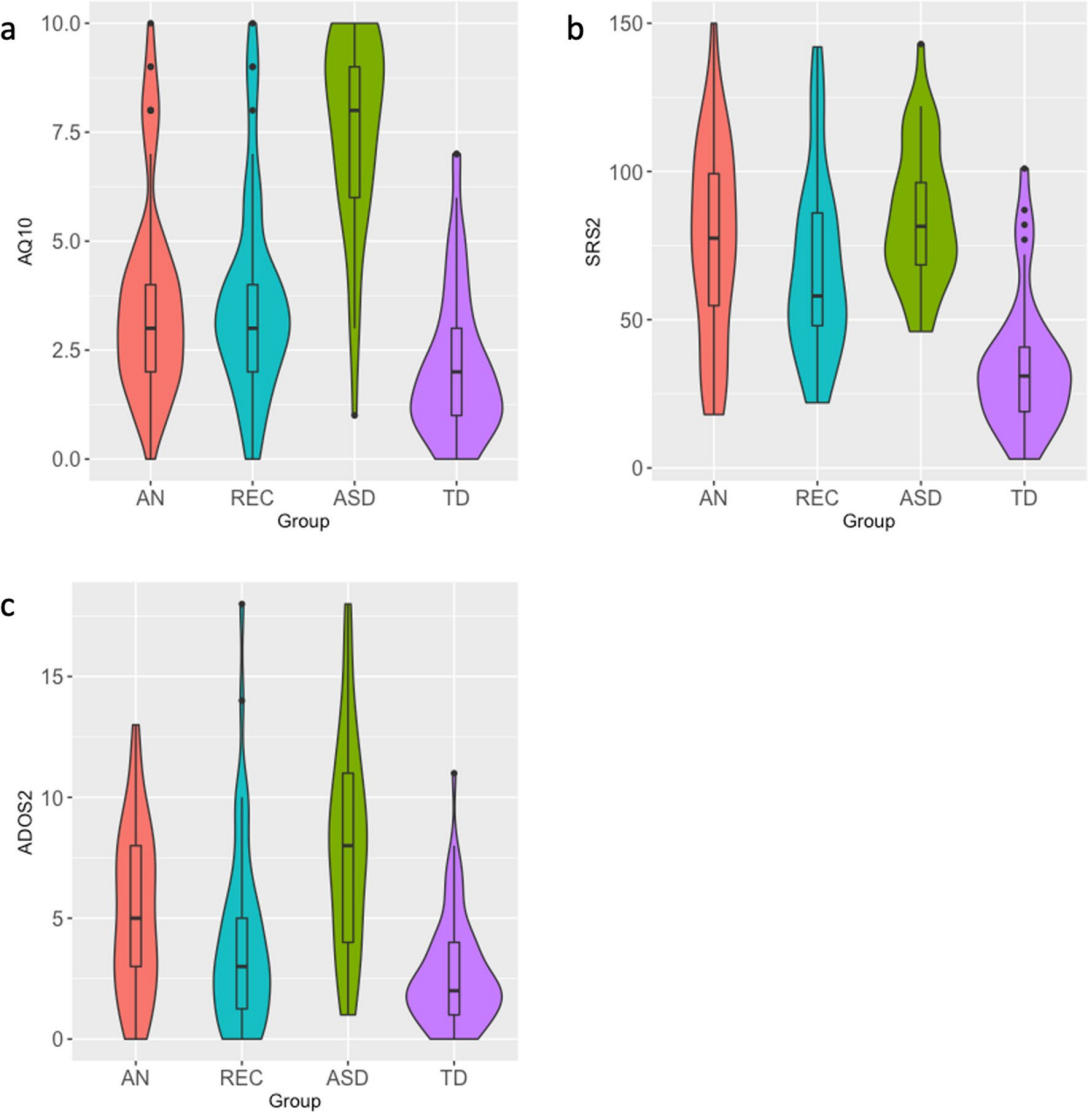
Violin plots showing the distribution of (**a**) AQ-10 (**b**) SRS-2 and (**c**) ADOS-2 total scores. Box plots show the median, interquartile range, minimum, and maximum scores within each group

Figure 1 shows the distribution of total scores on the AQ-10, SRS-2, and ADOS-2 for each group. Group differences in total scores, subscale scores, and the proportion of participants scoring above clinical cut-offs are provided in Table 2. Note that although Fig. 1 displays medians within boxplots, SRS-2 scores were normally distributed and means and standard deviations are presented in Table 2. As a small proportion of participants with ASD (*n* = 13) took the adolescent parent-report version of the AQ-10, we compared median scores in those who took the adult self-report (*M* = 7.50, IQR = 3.00) versus the adolescent parent-report version (*M* = 9.00, IQR = 2.50). There was no significant difference between groups, *U* = 232.50, *p* = 0.058. Thus, scores from both versions were analysed together. Median scores on the AQ-10 were higher in individuals with ASD compared to AN and REC, who did not significantly differ from one another, but scored significantly higher than TDs.

**Table 2:** Mean (SD)/median (IQR) total and subscale scores on ASD measures

	AN (*n* = 64)	REC (*n* = 46)	ASD (*n* = 41)	TD (*n* = 67)	Test statistics	*p*-value	Effect size
*AQ-10*							
Total^†^	3.00 (2.50)^a^	3.00 (2.00)^a^	8.00 (3.00)^b^	2.00 (2.00)^c^	*X*^2^(3) = 82.22	** < 0.001**	0.37
% scoring above cut-off (6)	14.8%^a^	18.2%^a^	84.6%^b^	4.6%^a^	*X*^2^(3) = 91.95	** < 0.001**	0.66
*SRS-2*^‡^							
Total (raw)	76.06 (33.39)^ab^	67.09 (30.48)^a^	84.92 (23.39)^b^	34.88 (22.85)^c^	*F*(3,137) = 24.02	** < 0.001**	0.35
Total (t-score)	62.66 (11.86)^ab^	59.33 (10.75)^a^	65.67 (8.30)^b^	48.03 (8.07)^c^	*F*(3,137) = 23.92	** < 0.001**	0.34
Social awareness	6.09 (3.41)^ac^	6.52 (3.28)^a^	9.81 (2.47)^b^	4.63 (2.81)^c^	*F*(3,137) = 19.83	** < 0.001**	0.30
Social cognition	13.28 (6.66)^a^	11.33 (5.56)^a^	17.67 (3.98)^b^	6.00 (5.25)^c^	*F*(3,137) = 30.41	** < 0.001**	0.40
Social communication	22.63 (11.46)^ab^	19.79 (12.10)^a^	28.58 (8.54)^b^	9.73 (8.77)^c^	*F*(3,137) = 22.63	** < 0.001**	0.33
Social motivation	17.81 (7.20)^a^	16.00 (7.16)^a^	14.67 (5.27)^a^	8.95 (4.75)^b^	*F*(3,137) = 14.63	** < 0.001**	0.24
Restricted interests and repetitive behaviours	16.25 (8.36)^a^	13.45 (7.27)^a^	14.19 (7.25)^a^	5.57 (4.19)^b^	*F*(3,137) = 17.68	** < 0.001**	0.28
*ADOS-2*							
Total^†^	5.00 (5.00)^a^	3.00 (4.25)^ac^	8.00 (7.00)^b^	2.00 (3.00)^c^	*X*^2^(3) = 50.69	** < 0.001**	0.22
Social affect^†^	3.50 (4.75)^a^	2.00 (3.00)^ac^	7.00 (7.00)^b^	1.00 (3.00)^c^	*X*^2^(3) = 47.58	** < 0.001**	0.21
Restricted and repetitive behaviour^†^	1.00 (200)^a^	0.00 (3.00)^ab^	1.00 (2.00)^ab^	0.00 (1.00)^b^	*X*^2^(3) = 12.69	**0.005**	0.05
% scoring above cut-off [8]	26.6%^a^	15.2%^ac^	56.1%^b^	3.0%^c^	*X*^2^(3) = 43.21	** < 0.001**	0.45

ADOS-2, autism diagnostic observation schedule, 2nd edition; AN, anorexia nervosa; ASD, autism spectrum disorder; AQ-10, 10-item autism quotient; IQR, interquartile range; REC, recovered anorexia nervosa; TD, typically developing controls; SD, standard deviation; SRS-2, Social Responsiveness Scale, 2nd edition

Different superscripts indicate significant differences between groups, significant *p*-values (< .01) are highlighted in bold

^†^Median and IQR

^‡^Note that 64% of the total sample took the SRS-2; 32 AN, 33 REC, 36 ASD, 40 TD

Total mean scores on the SRS-2 followed a similar pattern, however scores in the AN group did not significantly differ from ASD or REC, lying between the two. Regarding the mean SRS-2 *t* score, participants with AN and participants with ASD fell within the mild range, indicating clinically significant difficulties in social behaviour. Mean t scores in the REC and TD groups fell within the normal range. Individuals with ASD had the highest levels of social awareness, social cognition, and social communication symptoms on the SRS-2, followed by AN and REC, and TDs the lowest levels of symptoms. On the social motivation and restricted interests and repetitive behaviour subscales, AN, REC, and ASD all showed significantly higher scores than TD, but did not differ from one another. Individuals with ASD had significantly higher ADOS-2 total scores than the other three groups. AN had significantly higher scores than TD, while REC did not differ from AN or TD, with scores lying between the two groups. Social affect scores showed a similar pattern, however restricted and repetitive behaviours were only elevated in AN compared to TD.

Note that one of the AN studies did not include the SRS-2, and SRS-2 data from 5 participants with ASD was missing. Thus, scores were available for 64% (*n* = 141) of the total sample. In order to establish whether this subsample was similar on key demographic characteristics to the whole sample, independent samples t tests were run to compare age and IQ in those with SRS-2 data to those without. Participants with SRS-2 scores were significantly older (*M* = 23.00, SD = 3.91) than those without (*M* = 19.25, SD = 3.35), *t*(216) =  − 7.11, *p* < 0.001, however there were no significant differences in IQ. Group differences analyses for the ADOS-2 and AQ-10 were re-run in this subsample, to establish whether results remained the same as in the whole sample. Some significant differences between groups became non-significant, possibly due to the reduced sample size. However, effect sizes, as well as the distributions of scores remained similar to the whole sample. These results are presented in Additional File 2.

### ASD symptoms-item level

ADOS-2 items, with the proportion of participants endorsing each item, are displayed in Table 3.

**Table 3:** Proportion of participants endorsing each ADOS-2 item (non-zero scores)

	AN (*n* = 64) (%)	REC (*n* = 46) (%)	ASD (*n* = 41) (%)
*Language and communication*			
A1. Overall level of non-echoed spoken language	0	0	0
A2. Speech abnormalities^†^	20^a^**	13 ^a^**	46 ^b^**
A3. Immediate echolalia	2	2	0
A4. Stereotyped/idiosyncratic words or phrases^†^	22	20	17
A5. Offers information	31	30	29
A6. Asks for information	72	80	85
A7. Reporting of events	33	44	42
A8. Conversation^‡^	34	33	59
A9. Descriptive gestures	42	30	59
A10. Emphatic or emotional gestures^‡^	52 ^a^**	28 ^b^**	73 ^a^**
*Reciprocal social interaction*			
B1. Unusual eye contact^‡^	23	13	37
B2. Facial expressions^‡^	44	39	66
B3. Language production and linked nonverbal communication	19 ^a^**	13 ^a^**	64 ^b^**
B4. Shared enjoyment	32	33	39
B5. Communication of own affect^‡^	42	26	42
B6. Comments on others’ emotions/empathy	44 ^ab^**	24 ^b^**	66 ^a^**
B7. Insight into typical social situations/relationships^‡^	28	17	42
B8. Responsibility	11 ^a^**	4 ^a^**	37^b^**
B9. Quality of social overtures^‡^	16 ^a^**	11 ^a^**	54 ^b^**
B10. Amount of social overtures/maintenance of attention	30	33	49
B11. Quality of social response^‡^	16 ^a^**	20 ^a^**	66 ^b^**
B12. Amount of reciprocal social communication	33	41	61
B13. Overall quality of rapport^‡^	27 ^ab^*	20 ^b^*	49 ^a^*
*Creativity*			
C1. Imagination/creativity	39^a^**	33 ^a^**	78 ^b^**
*Stereotyped behaviours and restricted interests*			
D1. Unusual sensory interest^†^	48^a^*	30^ab^*	17^b^*
D2. Hand and finger and other complex mannerisms^†^	20	22	10
D3. Self-injurious behaviour	2	0	0
D4. Excessive interest in specific topics or repetitive behaviours	13	9	22
D5. Compulsions or rituals	13	9	5
*Other abnormal behaviours*			
E1. Overactivity/agitation	7	10	5
E2. Tantrums, aggression, negative or disruptive behaviour	0	0	2
E3. Anxiety	20	24	27

ADOS-2, autism diagnostic observation schedule, 2nd edition; AN, anorexia nervosa; ASD, autism spectrum disorder; REC, recovered anorexia nervosa

Different superscripts indicate significant differences (< .01) between groups

^*^p < 0.01; **p < 0.001

^†^Restricted and repetitive behaviour subscale items

^‡^Social affect subscale items

A significantly higher proportion of individuals with ASD endorsed six of the ADOS-2 items, compared to AN and REC: Speech abnormalities (A2), Language production and linked nonverbal communication (B3), responsibility (B8), quality of social overtures (B9), quality of social response (B11), and Imagination/creativity (C1). However, only one item, quality of social response (B11), adequately discriminated between AN and ASD (present in 66% of individuals with ASD, 16% of those with acute AN, and 20% of REC).

## Discussion

The aim of the current study was to compare profiles of ASD symptoms in adolescents and young women with ASD, AN, REC, and TD, on both self-report and clinical interview measures. Across measures, women with ASD showed the highest scores, and TD the lowest. Generally, individuals with AN and REC showed intermediate levels of ASD symptoms, scoring between the other two groups. This was not the case for restricted interests and repetitive behaviours however, where individuals with AN scored similarly to participants with ASD. We also aimed to examine group differences in individual ADOS-2 item endorsement, in order to provide a more detailed symptom profile in each of our clinical groups. Overall, many of the items endorsed by individuals with ASD were also apparent in individuals in both the acute and recovered stages of AN. However, a significantly higher proportion of individuals with ASD endorsed six of the items, compared to AN and REC, most of which concerned reciprocal social interaction. Only one item, quality of social response (B11), adequately discriminated between ASD and non-ASD participants.

Total scores on the AQ-10 were significantly higher in individuals with ASD compared to AN and REC, who did not differ from one another, but scored significantly higher than TD. This pattern was also found in a previous study that used the full 50-item version of the AQ in women with AN, men with ASD, and TD [39]. Although a scar-effect of the illness cannot be ruled out, the addition of a recovered group in the current study suggests that elevated AQ scores are not merely a result of starvation or other factors associated with the acute state of AN. Inspection of the violin plots shows clear differentiation between the two AN groups, ASD, and TD. While scores in those with ASD are concentrated at the higher end of the scale, scores in AN, REC, and TD are skewed towards the lower end, with a small number of outliers in the AN and REC groups showing high scores, similar to those with ASD. This suggests that the AQ-10 may be useful in screening for those with AN and possible ASD. Relatedly, the observation that 85% of participants with ASD scored above the clinical cut-off supports the use of the AQ-10 as a screening measure. This finding is consistent with recent reports showing the AQ-10 to be sensitive to female presentations of ASD [67, 68].

A somewhat similar pattern was found for SRS-2 total scores, with the ASD group showing the highest scores and the TD group showing the lowest. However, there was far less differentiation between the three clinical groups than was seen for the AQ-10; participants with AN did not significantly differ from those with ASD or REC. Indeed, previous research has suggested that although the SRS-2 is relatively accurate at differentiating ASD from typical development, specificity is much lower when differentiating ASD from other psychiatric populations. This is especially the case for psychiatric disorders with overlapping symptomatology, such as social anxiety disorder [69, 70]. For example, South and colleagues [71] found that adults with high levels of anxiety scored significantly higher on the SRS-2 than TD, but lower than individuals with ASD. On the other hand, the presence of an ASD diagnosis was not ruled out in the anxiety group, therefore elevated SRS-2 scores could represent genuine ASD symptoms or comorbidity. This is also the case in our study, where only those with a self-reported diagnosis of ASD could be excluded. As individuals with AN also present with high levels of social anxiety [22], attempts to differentiate between ASD and social anxiety symptoms is a particularly important challenge for clinical formulation and treatment planning in this population.

While there was some differentiation between the three clinical groups on most of the SRS-2 subscales, this was not the case for the social motivation and restricted interests and repetitive behaviours subscales, where AN, REC, and ASD displayed equally high scores compared to TD. Social motivation encompasses a variety of dispositions that bias humans to attend to social stimuli, seek and take pleasure from social interactions, and build social bonds. Disruptions in these processes are hypothesised to underlie social difficulties in ASD [72]. While social motivation has received less attention in eating disorder research, recent behavioural evidence complements our findings. Individuals with AN show reductions in social attention [73, 74], derive less pleasure from social interactions [75], and find social information less rewarding, compared to TD [76]. Whether similar brain processes underlie reduced social motivation in AN and ASD is an interesting question for future research. Alternatively, it has been suggested that the subscale actually captures social avoidance rather than social motivation [71]. Regardless, our findings suggest difficulties in this domain persist into recovery from AN and may represent a transdiagnostic feature of both AN and ASD.

Restricted and repetitive behaviours differed slightly across the SRS-2 and the ADOS-2. While participants with AN, REC, and ASD all scored significantly higher than TD on the SRS-2, only AN had significantly higher restricted and repetitive behaviour scores on the ADOS-2 compared to TD. This is likely in part due to differences in self-report versus clinical interview measures. In the ADOS-2, only behaviours that are observed during the course of the 40–50 min interview are coded, whereas the SRS-2 asks the participant to rate their behaviour over the past six months. Given that women with ASD often camouflage their symptoms [10], it is perhaps not surprising that they displayed relatively few restricted and repetitive behaviours during the interview. However, their scores on the SRS-2 would suggest that they experience inflexible behaviour, sensory sensitivities, and intense interests in their daily lives. It is also important to note that although scores were similar on this SRS-2 subscale across the three clinical groups, the motivations and aetiology of these behaviours may be different in AN and ASD. For example, fixation or anxiety around food may lead an individual with AN to score highly on item 28, “I think or talk about the same thing over and over”. In contrast, one might expect someone with ASD to score highly on this item due to an intense special interest. This issue is complicated even more in those with comorbid AN and ASD, with qualitative studies showing overlap between special interests and food restriction or exercise in women with both conditions [77].

Related to this point, although median scores on the ADOS-2 restricted and repetitive behaviours subscale were similar in AN, REC, and ASD, this appeared to be driven by different patterns of individual ADOS-2 item endorsement. A significantly higher proportion of women with ASD (46%) showed speech abnormalities associated with autism (A2) compared to both acute (20%) and recovered AN (13%). Conversely, unusual sensory interests (D1) were more common in acute AN (48%) compared to ASD (17%). This item refers to sensory seeking behaviour, for example in visual or textural aspects of the ADOS-2 materials, objects in the environment, or oneself (e.g., clothing). Our findings contrast with past questionnaire-based assessments, which have suggested that people with AN experience heightened sensory sensitivity (hypersensitivity), and find sensory experiences as aversive, resulting in avoidance [78, 79]. However, studies using objective measures are more mixed, with a recent review suggesting that individuals with AN show reduced taste sensitivity relative to controls [80].

Our findings suggest that difficulties in some aspects of reciprocal social interaction are more prevalent in ASD than AN or REC. In particular, quality of social response (ADOS-2 item B11), was found to be the best item for discriminating between AN and ASD. This may be an important area to assess when exploring suspected ASD in individuals with AN. Difficulties in social reciprocity are a core characteristic of ASD, but have not been closely studied in AN. In a qualitative study exploring friendship experiences in those with AN, participants highlighted issues with anxiety, social comparison and judgment from others, and exclusion, rather than difficulties in social reciprocity or social skills per se [24]. Although women with ASD also report similar difficulties with exclusion and anxiety, they also report frequent mistakes and misunderstandings of typical social conventions [23]. While patients with AN often appear withdrawn and socially anxious, it seems that only a minority of patients show difficulties in understanding the to and fro of conversation, or with responding appropriately to social prompts [64]. These individuals may indeed meet diagnostic criteria for ASD.

## Limitations

The current study has several limitations. Firstly, our study focussed on ASD symptoms in individuals with average of above average IQ. It is possible that symptoms may differ in autistic individuals with intellectual disability. Secondly, although participants with a self-reported diagnosis of ASD were excluded from the AN and REC groups, the original studies did not provide a full diagnostic assessment for ASD for participants in these groups, and participants with ASD were not assessed for eating disorders. This may have resulted in some diagnostic overlap between the AN/REC groups and the ASD group. As a result, symptoms could appear more similar across the clinical groups than they would if there was no diagnostic crossover. Relatedly, had the original studies included a developmental assessment of ASD symptoms, it would be possible to see which ASD symptoms may have been present before the onset of AN. This would provide important evidence regarding the aetiology of ASD symptoms in AN.

The cross-sectional design is another limitation of the current study. Past research has demonstrated that high levels of ASD symptoms in individuals with AN are associated with poorer outcomes [81, 82]. Therefore, it is possible that differences in ASD symptoms contributed to the recovery of the REC group. Longitudinal studies are required to examine whether ASD symptoms are stable over time in individuals with AN, or whether they are related to changes in clinical state. On the other hand, we did not require eating disorder psychopathology to be completely absent in REC participants for them to be included in the study. Therefore it could be argued that some individuals in this group were not fully recovered. However, no single set of criteria have been agreed upon to define recovery from AN, and the criteria used will partly depend on the research question. As our aim was to include a group of individuals with past AN that were no longer suffering from the acute effects of starvation (to ensure ASD symptoms were not a product of this), we emphasised a rather stringent weight criteria rather than the absence of eating disorder cognitions. Nonetheless, mean EDE-Q scores in the REC group were rather low, similar to previously published normative data for young women [83].

Finally, our study only included adolescent and young adult women, aged 12–30 years. Given that AN also occurs in middle and late adulthood [84], future research could include a wider age range in order to better represent this population. A final limitation is the reduced sample size in our SRS-2 group comparisons, as well as the use of the adolescent version of the AQ-10 in a small number of autistic participants. It could be argued that the differences seen between the SRS-2 and the other ASD symptom measures may be due to the older age of the subsample who took the SRS-2. However, distributions of scores and effect sizes on the AQ-10 and ADOS-2 were similar in this subsample as in the whole sample, providing evidence against this possibility. Nonetheless, replication of our findings in larger samples is required.

## Conclusions

To conclude, young women with AN show similarities to women with ASD on both self-report and clinical interview measures of ASD symptoms, albeit often in an attenuated form. Regarding the aetiology of ASD symptoms in AN, some clues are provided by our comparisons of AN and REC. Many of the symptoms seen in AN were also seen in REC, with no significant differences between groups on the AQ-10, SRS-2, and ADOS-2. This would suggest that ASD symptoms are not solely related to starvation or the acute state in AN. However, inspection of the distributions of scores shows that while differences were not significant, REC tended to score slightly lower than AN, and did not significantly differ from TD on the ADOS-2. Thus, our results suggest that ASD symptoms may be a combination of state and trait features in AN. Our findings may be useful for clinicians interested in identifying possible ASD comorbidity and adapting treatment for patients within eating disorder services.

## Supplementary Information

**Additional file 1**. Participant recruitment information.

**Additional file 2**. SRS-2 sub-sample analyses.

## Data Availability

The datasets analysed during the current study are not publicly available, but can be requested from the authors of the original studies.

## Abbreviations

AQ-10: 10-Item autism quotient
AQ: Autism quotient
AN: Anorexia nervosa
ANOVA: Analysis of variance
ADI-R: Autism diagnostic interview-revised
ADOS-2: Autism diagnostic observation schedule, 2nd edition
ASD: Autism spectrum disorder
BAI: Beck anxiety inventory
BDI: Beck depression inventory
BMI: Body mass index
BYI-II: Beck Youth Inventory, 2nd edition
DSM-IV: Diagnostic and Statistical Manual of Mental Disorders, 4th edition
DSM-5: Diagnostic and Statistical Manual of Mental Disorders, 5th edition
EDE-Q: Eating disorder examination questionnaire
HADS: Hospital Anxiety and Depression Scale
ICD-10: International Classification of Diseases, 10th Revision
IR: Interquartile range
IQ: Intelligence quotient
NART: National Adult Reading Test
REC: Recovered anorexia nervosa
SCID-5-RV: Structured Clinical Interview for DSM-5 Disorders, research version
SRS-2: Social Responsiveness Scale, 2nd edition
SD: Standard deviation
TD: Typically developing comparison group
WAIS: Wechsler Adult Intelligence Scale
WASI-II: Wechsler Abbreviated Scales of Intelligence, 2nd edition
WISC-III: Wechsler Intelligence Scale for Children, 3rd edition
