# Supplementary material for: Autism symptoms in anorexia nervosa: a comparative study with females with autism spectrum disorder
Source: Mol Autism. 2021 Jun 30;12:47. doi: 10.1186/s13229-021-00455-5 (PMC8247081; doi:10.1186/s13229-021-00455-5)
Supplement: Supplementary file 1 — Additional file 1. Participant recruitment information. [file 13229_2021_455_MOESM1_ESM.docx]

**Additional file 1**

*Participant recruitment*

The EU-AIMS Longitudinal European Autism Project is a multi-site study across six European specialist ASD centres: Institute of Psychiatry, Psychology and Neuroscience, King’s College London (IoPPN/KCL, United Kingdom), Autism Research Centre, University of Cambridge (UCAM, United Kingdom), University Medical Centre Utrecht (UMCU, Netherlands), Radboud University Nijmegen Medical Centre (RUNMC, Netherlands), Central Institute of Mental Health (CIMH, Germany) and the University Campus Bio-Medico (UCBM) in Rome, Italy. Participants with ASD were recruited from a variety of sources including existing volunteer databases, existing research cohorts, clinical referrals from local outpatient centres, special needs schools, mainstream schools and local communities. Baseline recruitment took place between January 2014 and March 2017. At each site an independent ethics committee approved the study. All participants and their parent/carer or legal guardian (for those under 18 years) provided written informed consent.

Participants with AN, REC, and TD came from two studies investigating social and emotional functioning in AN. Both studies took place in the same laboratory at the IoPPN, KCL, and followed broadly similar recruitment strategies. One of the studies was cross-sectional (26); recruitment took place between February 2018 and June 2019. The other study (37) was longitudinal but only baseline data was used; baseline recruitment took place between June 2017 and April 2019. AN, REC, and TD participants were recruited through KCL staff and student notices, advertisements in the local community, and online (B-eat, call for participants, and MQ mental health). Participants with AN were also recruited from two NHS specialist eating disorder services: South London and Maudsley trust and Central and North West London trust. Each study received approval from an independent ethics committee, and all participants and their parent/carer (for those under 18 years) provided written informed consent.
