# Supplementary material for: Autism symptoms in anorexia nervosa: a comparative study with females with autism spectrum disorder
Source: Mol Autism. 2021 Jun 30;12:47. doi: 10.1186/s13229-021-00455-5 (PMC8247081; doi:10.1186/s13229-021-00455-5)
Supplement: Supplementary file 2 — Additional file 2. SRS-2 sub-sample analyses. [file 13229_2021_455_MOESM2_ESM.docx]

**Additional file 2**

| Table S1. Median (IQR) total and subscale scores, and proportion scoring above cut-off on ASD measures in the subgroup of participants with SRS-2 data | | | | | | | |
| --- | --- | --- | --- | --- | --- | --- | --- |
|  | AN (n = 32) | REC (n = 33) | ASD (n = 36) | TD (n = 40) | Test statistics | p-value | ﻿Effect size |
| *AQ-10* |  |  |  |  |  |  |  |
| Total | 3.00 (4.50)^a^ | 4.00 (2.00)^a^ | 8.00 (3.00)^b^ | 1.00 (1.75)^c^ | X^2^(3) = 62.17 | **<.001** | 0.44 |
| % scoring above cut-off (6) | 25.0%^a^ | 22.6%^a^ | 83.3%^b^ | 5.0%^a^ | X^2^(3) = 57.14 | **<.001** | 0.64 |
| *ADOS-2* |  |  |  |  |  |  |  |
| Total | 3.50 (4.75)^ab^ | 2.00 (4.00)^a^ | 8.00 (6.75)^b^ | 2.00 (3.00)^a^ | X^2^(3) = 36.15 | **<.001** | 0.24 |
| Social affect | 3.00 (4.75)^ab^ | 2.00 (4.00)^a^ | 7.50 (6.50)^b^ | 2.00 (2.00)^a^ | X^2^(3) = 28.99 | **<.001** | 0.19 |
| Restricted and repetitive behaviour | 0.00 (1.00)^ab^ | 0.00 (0.00)^a^ | 1.00 (2.00)^b^ | 0.00 (0.00)^a^ | X^2^(3) = 28.77 | **.001** | 0.19 |
| % scoring above cut-off (8) | 18.8%^a^ | 21.2%^a^ | 58.3%^b^ | 2.5%^a^ | X^2^(3) = 33.21 | **<.001** | 0.49 |
| Different superscripts indicate significant differences between groups, significant p-values are highlighted in bold. | | | | | | | |


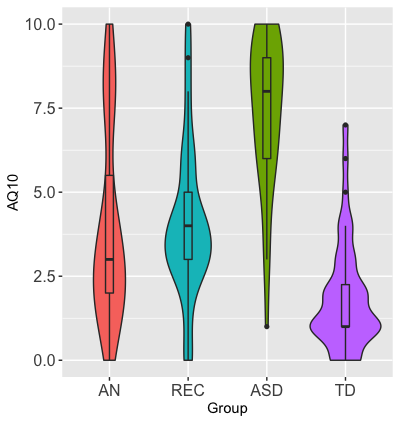

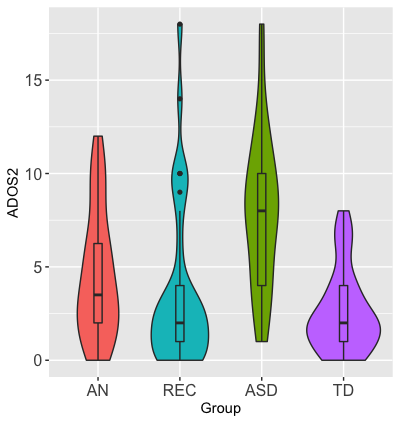


Figure S1. Violin plots showing the distribution of AQ-10 (left) and ADOS-2 (right) total scores in the reduced sample with SRS-2 data. Box plots show the median, interquartile range, minimum, and maximum scores within each group.
